# Adverse Experiences, Protective Factors, and Obesity in Latinx and Hispanic Youths

**DOI:** 10.1001/jamanetworkopen.2025.47104

**Published:** 2025-12-04

**Authors:** Victoria Goldman, Sevan Esaian, Miguel Ángel Rivas Fernández, Jonatan Ottino Gonzalez, Nicole Karcher, Jeffrey I. Gold, Alaina P. Vidmar, Shana Adise

**Affiliations:** 1 Department of Pediatrics, Division of Endocrinology, Diabetes and Metabolism, Children’s Hospital Los Angeles, Los Angeles, California; 2 Area of Developmental and Educational Psychology, Department of Psychology, Sociology and Philosophy, University of León, León, Spain; 3 Department of Psychiatry, Washington University School of Medicine in St Louis, St Louis, Missouri; 4 Departments of Anesthesiology, Pediatrics, and Psychiatry & Behavioral Sciences, Keck School of Medicine of the University of Southern California, Los Angeles; 5 Department of Anesthesiology Critical Care, The Saban Research Institute at Children’s Hospital Los Angeles, Los Angeles, California; 6 Department of Nutritional Sciences, University of Georgia, Athens

## Abstract

**Importance:**

Pediatric obesity rates are rising, and adverse childhood experiences (ACEs) may contribute by promoting stress-induced weight gain. Few studies have examined the association of ACEs with body mass index (BMI) and youth-reported protective factors, particularly among Latinx and Hispanic youths, who face higher rates of ACEs and obesity.

**Objective:**

To evaluate the association of ACEs with BMI in a population-based cohort and determine whether youth-reported protective factors (eg, self-coping skills, caregiver or friend support, or overall support) moderate this association, with a focus on Latinx and Hispanic youths.

**Design, Setting, and Participants:**

This large, cross-sectional study of youths aged 11 to 12 years was conducted using year 2 data (July 2018 to March 2020) of the Adolescent Brain Cognitive Development (ABCD) study, a prospective, 10-year longitudinal, 21-site dataset comprised of a population-cohort of US youths. Data were analyzed between August 2024 and March 2025.

**Exposures:**

Cumulative ACEs (12 categories) and youth-reported protective factors (4 categories).

**Main Outcome and Measures:**

The primary outcome was the ability of self-reported protective factors to moderate the hypothesized association of ACEs with BMI in Latinx and Hispanic youths. Linear mixed-effects models explored associations of ACEs, protective factors, and ethnicity (ie, Latinx and Hispanic or non-Hispanic) with BMI, while controlling for confounders (eg, sex, age, puberty, and socioeconomic status).

**Results:**

There were 5435 youths with available data at ages 11 to 12 years (1141 Latinx and Hispanic [21.0%]; 2636 female [48.5%]; mean [SD] age, 143.1 [7.6] months). Compared with non-Hispanic youths, Latinx and Hispanic youths had greater BMI (mean [SD], 22.1 [5.0] vs 20.3 [4.6]; *P* < .001) and more ACEs (mean [SD], 2.1 [1.7] vs 1.7 [1.7]; *P* < .001). Across all youths, ACEs were significantly associated with BMI, with a 0.431 BMI increase for every 1.7-point increase (1 SD) in ACE score. In Latinx and Hispanic youths only, self-coping (β = −0.74; 95% CI, −1.03 to −0.46; *P* < .001), caregiver support (β = −0.38; 95% CI, −0.66 to −0.11; *P* = .006), and overall protective score (β = −0.55; 95% CI, −0.61 to −0.06; *P* < .001) moderated the association of ACEs with BMI.

**Conclusions and Relevance:**

These findings highlight the clinical importance of early ACE screening to identify at-risk youths for targeted, trauma-informed weight management interventions, and gather support for the cultivation of resiliency-focused skills like self-coping and caregiver support. Pediatric obesity is a pressing public health issue, and these strategies hold potential to alter weight trajectories, which may improve health outcomes and reduce health disparities.

## Key Points

**Question:**

Do youth self-reported protective factors moderate the association of adverse childhood experiences (ACEs) with body mass index (BMI) in Latinx and Hispanic youths?

**Findings:**

In this cross-sectional study of 5435 youths from the Adolescent Brain Cognitive Development (ABCD) study, ACEs were associated with increased BMI. Youth-reported self-coping skills and perceived caregiver support moderated the association of ACEs with BMI among Latinx and Hispanic youths, who had a greater number of ACEs and higher BMI than non-Hispanic youths.

**Meaning:**

These findings suggest that ACEs may increase youth obesity risk, but promotion of resiliency-focused skills may help improve pediatric weight trajectories among disproportionately impacted populations.

## Teaser

This cross-sectional study analyzes the association of adverse childhood experiences with obesity among Latinx and Hispanic youths and examines the ability of protective factors to moderate this association.

## Introduction

Pediatric obesity rates continue to rise, affecting approximately 20% of youths in the US, with the highest prevalence among Latinx and Hispanic populations (approximately 26%).^1,2,3^ Amid the multifaceted contributors to childhood obesity, chronic severe stress may be one factor,^4,5,6,7^ exerting influence through hormonal dysregulation and altered food intake.^8,9,10^ Substantial stress during childhood is becoming increasingly common, as 1 in 2 youths are affected by adverse childhood experiences (ACEs; ie, stressful or traumatic events such as sexual abuse), with rates further elevated in Latinx and Hispanic communities.^11,12,13,14^ ACEs have been associated with poor health outcomes in adults, including cardiovascular disease, mental illness, obesity, and overall mortality^4,15,16,17,18^; yet, the association in youths remains less understood, with even fewer studies on individuals from historically underrepresented backgrounds.^19,20,21^ Furthermore, little is known about what self-reported protective factors may moderate the negative consequences of ACEs on weight. Given the high prevalence of pediatric obesity and ACEs in Latinx and Hispanic communities,^2,3,11,12,13,14^ it is imperative to deepen understanding of associations of ACEs with weight across populations at higher risk. Exploring both risk and protective factors may help identify strategies to moderate this association, improving health outcomes.

The connection between stress and weight gain is rooted in biological mechanisms. Acutely, stress hormones such as cortisol mobilize glucose, suppress digestion, and elevate blood pressure to prepare the body for physical threats (eg, fight or flight).^22,23^ While this may initially decrease appetite, the resulting energy demands ultimately prompt food consumption to replenish lost stores.^24,25^ Historically, physical threats were more prevalent, but many present-day stressors are psychological. Psychological stressors mobilize energy without expenditure and, with chronic exposure, can promote weight gain.^25^ Furthermore, chronic stressors can dysregulate the hypothalamic-pituitary-adrenal axis, triggering hypercortisolism and contributing to weight gain through insulin resistance, metabolic syndrome, sleep dysregulation, and altered eating behaviors.^8,10,18,24,26,27,28^ ACEs, often associated with chronic stress, exemplify how early life adversity can have lasting effects on weight gain throughout the lifespan.

Youths in Latinx and Hispanic communities are more likely to experience ACEs^11,12,13,14^ and high obesity rates.^2,3^ Therefore, it is possible that ACEs are contributing to obesity risk at an elevated rate in Latinx and Hispanic youths. This risk may stem from factors such as higher likelihood of living in historically unsafe neighborhoods, food insecurity, discrimination, and bullying.^2,13,29,30,31,32,33^ Despite the significance of these health disparities, limited research has examined the association of ACEs with body mass index (BMI) within this population. As obesity rates continue to rise among Latinx and Hispanic youths,^2,3^ it is crucial to consider unique factors that may exacerbate obesity risk to help develop community-specific, targeted interventions.

Several studies have suggested that protective factors like coping skills and supportive relationships or environments may moderate the harmful consequences of ACEs on health.^34,35,36,37^ Among adults with childhood adversity, greater resiliency, positive experiences, and supportive parental relationships have been associated with improved health.^34,35,38,39^ However, less is known about these associations in children, particularly among high-risk groups, like Latinx and Hispanic youths. In part, this may be due to the fact that studies have often focused on small or nongeneralizable samples (eg, singular clinic),^40,41,42,43^ family-reported anthropometrics,^6,44,45,46,47,48^ and/or parent-reported protective factors (see the eAppendix and eTable 1 in Supplement 1 for an overview of these studies).^6,40,41,43,44,45,46,48,49,50^ Moreover, even in the absence of these methodological criteria, studies have not examined whether self-reported protective factors moderate the association of ACEs with obesity in Latinx and Hispanic youths, a population disproportionately impacted by both, yet understudied.^2,3,12,13,19^

The current study explored the extent to which self-reported protective factors moderated the association of ACEs with BMI among early adolescents. Analyses focused on adolescents aged 11 to 12 years from the population-based Adolescent Brain Cognitive Development (ABCD) study, with an emphasis on Latinx and Hispanic youths given their elevated rates of ACEs and obesity.^3,12,13,14^ Youth-reported protective factors were subsequently examined as potential moderators of this association. It was hypothesized that greater ACE exposure would be associated with higher BMI, with a larger-magnitude association among Latinx and Hispanic youth. Additionally, youth-reported protective factors were expected to attenuate the association of ACEs with BMI. Findings from this study may advance the field by adding insight into whether protective factors moderate the association of ACEs and BMI among Latinx and Hispanic youths. Understanding these associations is essential for informing the development of culturally informed prevention and intervention strategies in communities with heightened risk.

## Methods

### Study Design

This cross-sectional study used data from the ABCD Study, a large (11 878 participants at baseline), demographically diverse (21 sites; approximately 24% Latinx and Hispanic participants), prospective, 10-year longitudinal study in the US. It started in September 2016 to October 2018 when youths were aged 9 to 10 years. Caregivers and youths provided written informed consent and assent in accordance with the centralized institutional review board at the University of California San Diego, which approved this analysis. All 21 sites also received local institutional review board approval. This study used data from the 5.0 release.^51^ Only data collected prior to the COVID-19 pandemic (March 15, 2020) were included, given delayed or remote assessments. Analyses, conducted between August 2024 and March 2025, focused on the 2-year follow-up data when the most ACE-focused questions were collected. Reporting followed the Strengthening the Reporting of Observational Studies in Epidemiology (STROBE) reporting guideline for cross-sectional studies.

### Exclusion and Inclusion

The ABCD Study applied narrow exclusion criteria (see the eMethods in Supplement 1) to enhance population-level relevance, increase power, and reduce potential biases. To enhance analytic validity, additional exclusion criteria were applied for youths with (1) potential weight-impacting medications (eg, stimulants or insulin); (2) siblings (independence issues); (3) missing key demographic, ACEs, or protective factor data; and (4) invalid anthropometrics (eg, shrinking heights) (additional details in eMethods and eTable 2 in Supplement 1).

### Demographics

Caregivers reported their education level and the youth’s ethnicity, race, date of birth, and sex on surveys. Non-Hispanic or Hispanic/Latino/Latina could be selected by the caregiver for ethnicity, and race categories included American Indian, Alaska Native, Native Hawaiian, or Pacific Islander; Asian, Black; White; multiracial (ie, >1 race); and other (as selected by caregiver; additional details in the eMethods in Supplement 1). Due to potential inaccuracies in reported income, caregiver education was used as a proxy for socioeconomic status.

### Physical Data

Puberty was calculated using averaged youth and caregiver reports to the Pubertal Development Scale.^52^ In-person height and weight measurements were assessed by a trained researcher and converted into BMI (calculated as weight in kilograms divided by height in meters squared) and percentiles^53^ for clinical relevance (additional details in the eMethods in Supplement 1).

### ACEs

ACEs have traditionally been measured using a binary (yes or no) questionnaire of 10 ACEs (eg, sexual abuse or neglect) developed in the 1990s.^54^ Subsequently, additional ACEs (eg, bullying, neighborhood violence, and socioeconomic hardship) have been associated with poor health outcomes.^6,44,55,56,57,58,59^ Therefore, the original categories were expanded upon to include 12 categories of stressful or traumatic experiences: physical abuse, sexual abuse, neglect, emotional abuse, family drug or substance misuse, family mental health challenges, domestic violence, divorce, incarcerated caregiver, bullying, neighborhood violence, and basic needs insecurity. Because the ABCD Study did not have a singular ACE questionnaire, several questionnaires (eg, Life Events^60^ or Neighborhood Safety/Crime Survey^61^; see the eMethods in Supplement 1 for more details) were utilized. Youth responses were prioritized and supplemented with caregiver responses from surveys. Positive responses from baseline or 1-year follow-up were carried forward to account for wording differences and capture adversities across childhood. A positive item within a category was scored as 1 point for that category, regardless of how many items were endorsed. Category scores were then summed to a total ACE score (see eTable 3 in Supplement 1 for included ACE questions).

### Protective Factors

Four protective factors were derived from youth self-reports to 3 surveys^62,63,64^ that assessed (1) self-coping skills, (2) perceived caregiver warmth and support, and (3) friend support. An overall (fourth) protective factor score was calculated as the mean of the 3 scores, allowing insight into whether the combination of protective factors is needed to moderate risk. Protective factor questions are detailed in eTable 4 in Supplement 1.

### Statistical Analysis

Analyses were conducted using Python 3.11.8 (Python Software Foundation). Data were assessed for skewness, outliers, and multicollinearity among continuous fixed effects. Linear mixed-effects models were conducted using the Pymer4 statistics library^65^ to explore associations of ACE total score with raw BMI using cross-sectional data, while controlling for confounders (eg, puberty, sex, age, and caregiver education [proxy for socioeconomic status]) that are known to affect BMI.^66,67^ Continuous variables (eg, age and puberty) were standardized, and categorical variables (eg, sex and caregiver education) were effects-coded; site was a random effect.^68^

To confirm the literature, analyses first evaluated the association of ACEs with BMI in the overall sample. Given prior research demonstrating higher ACEs and BMI in Latinx and Hispanic youths,^2,3,12,13,14^ a 2-way interaction between ACEs and ethnicity (ie, Latinx and Hispanic vs non-Hispanic) on BMI was examined. Next, to assess whether protective factors moderated this association, a 3-way interaction between ACEs, ethnicity, and protective factors on BMI was conducted. Finally, to enhance clinical applicability, we examined individual ACEs to determine whether specific ACEs were influenced by protective factors. The Benjamini-Hochberg procedure^69^ was used to control the false discovery rate. Significance was defined as a 2-sided *P* < .05.

## Results

There were 5435 youths that met inclusion criteria (1141 Latinx and Hispanic [21.0%]; 4294 non-Hispanic [79.0%]; 2636 female [48.5%]; mean [SD] age, 143.1 [7.6] months; mean [SD] BMI, 20.65 [4.8] 1864 with overweight or obesity [34.4%]) (Table 1). Compared with non-Hispanic youths, Latinx and Hispanic youths had higher BMI (mean [SD], 22.1 [5.0] vs 20.3 [4.6]; *P* < .001) and rates of overweight and obesity (547 youths [47.9%] vs 1327 youths [30.9%]; *P* < .001) (eFigure 1 in Supplement 1).

**Table 1.** Youth Demographics

Characteristic	Participants, No. (%)			*P* value^a^
	Overall (N = 5435)	Latinx or Hispanic (n = 1141)	Non-Hispanic (n = 4294)	
Sex				
Female	2636 (48.5)	561 (49.2)	2075 (48.3)	.64
Male	2799 (51.5)	580 (50.8)	2219 (51.7)	
Age, mean (SD), mo	143.1 (7.6)	142.6 (7.8)	143.2 (7.6)	.02
Pubertal Developmental Scale score, mean (SD)^b^	2.8 (0.99)	2.9 (0.97)	2.7 (0.99)	<.001
BMI, mean (SD)^c^	20.7 (4.8)	22.1 (5.0)	20.3 (4.6)	<.001
Weight status (BMI percentiles)^d^				
Underweight (<5.0)	183 (3.4)	19 (1.7)	164 (3.8)	<.001
Healthy weight (5.0-84.9)	3378 (62.2)	575 (50.4)	2803 (65.3)	
Overweight (85.0-94.9)	889 (16.5)	232 (20.3)	667 (15.5)	
Obesity (≥95.0)	975 (17.9)	315 (27.6)	660 (15.4)	
Caregiver education				
<High school	238 (4.4)	151 (13.2)	87 (2.0)	<.001
High school or Generalized Education Degree	449 (8.3)	183 (16.0)	266 (6.2)	
Some college	1334 (24.5)	386 (33.8)	948 (22.1)	
Bachelor’s degree	1450 (26.7)	217 (19.0)	1233 (28.7)	
Postgraduate degree	1964 (36.1)	204 (17.9)	1760 (41.0)	
Race^e^				
American Indian, Alaska Native, Native Hawaiian, or Pacific Islander	41 (0.75)	15 (1.3)	26 (0.61)	<.001
Asian	135 (2.5)	13 (1.1)	122 (2.8)	
Black	681 (12.5)	32 (2.8)	649 (15.1)	
White	3635 (66.9)	652 (57.1)	2983 (69.5)	
Multiracial	625 (11.5)	154 (13.5)	471 (11.0)	
Other	245 (4.5)	78 (2.8)	27 (0.6)	
Missing	73 (1.3)	57 (5.0)	16 (0.4)	
Annual income, $				
50 000	1193 (22.0)	454 (39.8)	739 (17.2)	<.001
50 000-100 000	1397 (25.7)	320 (28.0)	1077 (25.1)	
>100 000	2454 (45.2)	242 (21.2)	2212 (51.5)	
Missing	391 (7.2)	125 (11.0)	266 (6.2)	

Abbreviation: BMI, body mass index.

^a^ *P* values reflect χ^2^ and *t* tests where appropriate.

^b^ Puberty was categorized based on the Pubertal Developmental Scale (see the eMethods in Supplement 1).

^c^ Calculated as weight in kilograms divided by height in meters squared and used as a continuous variable for analyses.

^d^ Weight status, categorized according to the Centers for Disease Control and Prevention BMI-for-age percentiles^53^

^e^ Race and ethnicity were reported by the caregiver. Multiracial (endorsement of more than 1 race) and other were race categories selected by the caregiver for responses not otherwise specified.

### ACE Prevalence

Youths reported experiencing 0 to 9 ACEs (max score 12) with a mean (SD) ACE score of 1.8 (1.7) (Table 2). Moreover, 1379 youths (25.4%) reported 0 ACEs, whereas 4056 youths (74.6%) reported at least 1 ACE, with 814 (15.0%) reporting 4 or more (see eFigure 2 in Supplement 1 for distribution by weight class). The most common ACEs were domestic violence (2013 youths [37.0%]), divorce (1441 youths [26.5%]), and basic needs insecurity (1272 youths [23.4%]). Latinx and Hispanic youths had higher mean (SD) ACE scores (2.1 [1.7]) than non-Hispanic youths (1.7 [1.7]) (*P* < .001). Only 187 Latinx and Hispanic youths (16.4%) reported 0 ACEs, compared with 1192 non-Hispanic youths (27.8%) (*P* < .001). Given increased ACEs and BMI in Latinx and Hispanic youths, the association between these factors was examined both in the overall youth sample and by ethnicity (ie, Latinx and Hispanic or non-Hispanic) to better understand and address potential disparities.

**Table 2.** Descriptive Statistics for ACEs^a^

ACE characteristic	Participants, No. (%)			*P* value^b^
	Overall (N = 5435)	Latinx or Hispanic (n = 1141)	Non-Hispanic (n = 4294)	
No. of ACEs				
0	1379 (25.4)	187 (16.4)	1192 (27.8)	<.001
1	1476 (27.2)	270 (23.7)	1206 (28.1)	.003
2	1088 (20.0)	279 (24.5)	809 (18.8)	<.001
3	678 (12.5)	200 (17.5)	478 (11.1)	<.001
4	384 (7.1)	102 (8.9)	282 (6.6)	.007
5	244 (4.5)	50 (4.4)	194 (4.5)	.91
6	111 (2.0)	34 (3.0)	77 (1.8)	.02
7	51 (0.9)	14 (1.2)	37 (0.9)	.34
8	19 (0.3)	5 (0.4)	14 (0.3)	.77
9	5 (0.1)	0	5 (0.1)	.55
ACE category				
Physical abuse	62 (1.1)	14 (1.2)	48 (1.1)	.88
Sexual abuse	122 (2.2)	23 (2.0)	99 (2.3)	.64
Neglect	324 (6.0)	82 (7.2)	242 (5.6)	.06
Emotional abuse	41 (0.8)	9 (0.8)	32 (0.8)	>.99
Family substance use	1005 (18.5)	238 (20.9)	767 (17.9)	.02
Family mental health	861 (15.8)	197 (17.3)	664 (15.5)	.15
Domestic violence	2013 (37.0)	435 (38.1)	1578 (36.8)	.41
Divorce	1441 (26.5)	396 (34.7)	1045 (24.3)	<.001
Incarcerated caregiver	511 (9.4)	120 (10.5)	391 (9.1)	.16
Bullying	1027 (18.9)	257 (22.5)	770 (17.9)	.001
Basic needs insecurity	1272 (23.4)	381 (33.4)	891 (20.8)	<.001
Neighborhood violence	983 (18.1)	276 (24.2)	707 (16.5)	<.001

Abbreviation: ACEs, adverse childhood experiences.

^a^ Further information on ACE questions can be found in eTable 3 in Supplement 1.

^b^ *P* values reflect χ^2 ^tests.

### Association of ACEs With BMI

**Figure 1.**
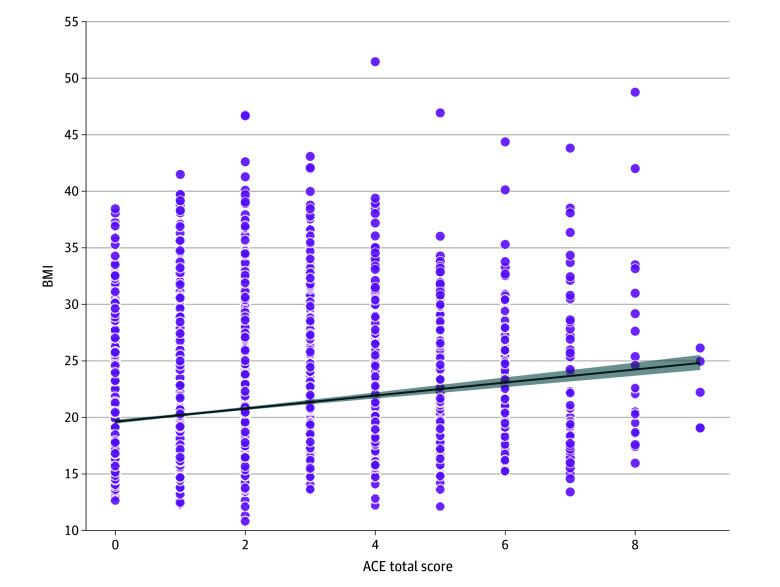
Association of Adverse Childhood Experiences (ACEs) Total Score With Body Mass Index (BMI) Data points represent individual BMI values for the overall sample (5435 participants) plotted against the ACE score, with the solid line indicating the fitted regression line and shaded area denoting the 95% CI. ACE scores ranged from 0 to 12.

Across all youths, ACEs were significantly associated with higher BMI. For every 1.7-point increase (equivalent to 1 SD) in ACE score, BMI increased by 0.431 (β = 0.43, 95% CI, 0.30 to 0.57; *P* < .001), while controlling for the aforementioned factors (eg, age, puberty, sex, and caregiver education) (Figure 1). The 2-way interaction between ACEs and ethnicity (ie, Latinx and Hispanic and non-Hispanic) was not significant (*P *for interaction = .62).

### Moderating Associations With Protective Factors

**Figure 2.**
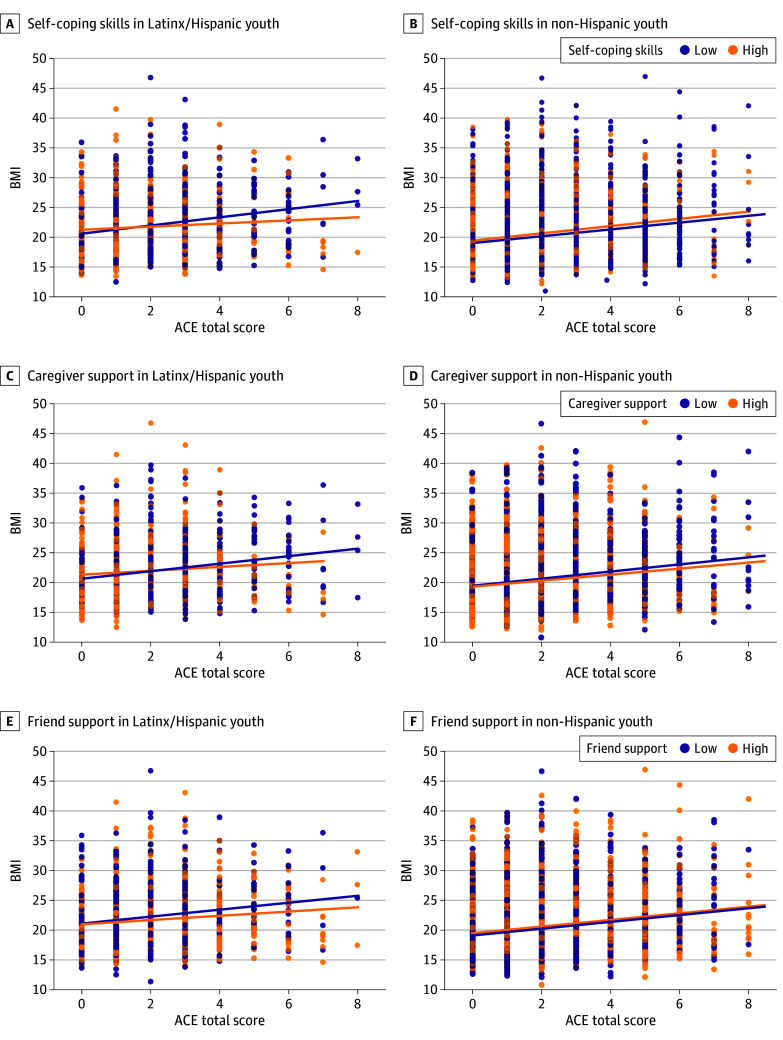
Association of Adverse Childhood Experiences (ACEs) and Protective Factors With Body Mass Index (BMI) by Ethnicity Data points represent individual BMI values plotted against the ACE score. Solid lines represent fitted regression associations stratified (for visual purposes only) by protective factor group. Protective factors were categorized into 2 groups based on median values: low (small-magnitude associations; dark blue) and high (large-magnitude associations; orange). ACE score ranged from 0 to 12.

Across the entire sample, there were no significant 2-way interactions between ACEs and protective factors (eg, self-coping, caregiver support, friend support, and overall protective score) on BMI. Significant 3-way interactions emerged between ACEs × ethnicity × protective factors on BMI. For Latinx and Hispanic youths only, self-coping (β = −0.74; 95% CI, −1.03 to −0.46; *P* < .001), caregiver support (β = −0.38; 95% CI, −0.66 to −0.11; *P* = .006), and overall protective score (β = −0.55; 95% CI, −0.61 to −0.06; *P* < .001) moderated the association of ACEs with BMI (Figure 2). Friend support was not significant. Pairwise combinations of protective factors (eg, self-coping skills and caregiver support mean score) are described in the eResults in Supplement 1.

Given the smaller proportion of Latinx and Hispanic youths than non-Hispanic youths, a sensitivity analysis stratified by ethnicity (2-way interaction; ie, ACEs × protective factor on BMI) was conducted to confirm results. Among Latinx and Hispanic youths, self-coping (β = −0.65; 95% CI, −0.92 to −0.38; *P* < .001), caregiver-support (β = −0.34; 95% CI, −0.61 to −0.08; *P* = .01), and overall protective score (β = −0.50; 95% CI, −0.63 to −0.01; *P* < .001) moderated the association of ACEs with BMI but not in non-Hispanic youths (eTable 5 in Supplement 1).

### Individual ACEs and Protective Factors

To enhance clinical relevance, analyses examined whether protective factors moderated the association of ACEs with BMI among Latinx and Hispanic youths. Self-coping (β = −2.45; 95% CI, −3.30 to −1.57; *P* < .001) and overall protective score (β = −1.50; 95% CI, −2.39 to −0.60; *P* = .001) attenuated the association of having an incarcerated caregiver (individual ACE) with BMI in Latinx and Hispanic youths (eTable 6 and eTable 7 in Supplement 1).

## Discussion

This cross-sectional study highlights the importance of protective factors in Latinx and Hispanic youths in moderating the association of ACEs with BMI. Specifically, self-coping, caregiver support, and overall support attenuated the association of ACEs with BMI in Latinx and Hispanic youths only, but not in the entire sample. This distinction suggests that protective factors may buffer adversity differently across groups, shaped by sociocultural and economic contexts. To date, few studies have focused on Latinx and Hispanic youths, and none, to our knowledge, have simultaneously incorporated youth self-reported protective factors with objectively measured anthropometric data in a large, sociodemographically diverse pediatric cohort. These findings highlight the need for trauma-informed and culturally sensitive interventions to address pediatric obesity, particularly in communities disproportionately impacted by ACEs and obesity.

Importantly, nearly 75% of youths reported experiencing ACEs, which exceeded commonly reported rates of approximately 50%.^26^ The most common ACEs were domestic violence, divorce, and basic needs insecurity. Higher rates may reflect self-reporting because ACE prevalence is often higher when reported by adolescents rather than caregivers.^70^ These estimates were even higher among Latinx and Hispanic (1 or more ACEs, 83.6%; obesity, 47.9%) compared with non-Hispanic youths (1 or more ACES, 72.1%; obesity, 30.9%), which is consistent with prior studies.^2,3,11,12,13,14^ In descending order of prevalence, divorce, basic needs insecurity, neighborhood violence, bullying, and family substance use were significantly more prevalent among Latinx and Hispanic youths. Increased ACE prevalence among Latinx and Hispanic youths may compound health disparities, underscoring the importance of identifying protective factors for high-risk populations.

Although ACEs were associated with higher BMI across the whole sample, protective factors, including self-coping skills, caregiver support, and overall support, buffered this association in Latinx and Hispanic youths only. Interestingly, when evaluating individual ACEs, self-coping, and overall protective score attenuated the association of caregiver incarceration with BMI. One potential reason that we did not find these associations for all youths may be related to cultural variation in stress exposure, coping strategies, cultural expectations, or environments. Although not determinative, sociocultural backgrounds likely shape stress experiences and acceptability or accessibility of protective factors.^71,72,73,74^ For example, a study in adults showed that those with lower incomes reported greater reliance on coping strategies than those with higher incomes.^75^ The greater economic hardship among Latinx and Hispanic youths compared with non-Hispanic White peers,^76^ may increase chronic stress exposure, limit resource access, or heighten the roles of protective factors.^75^ non-Hispanic youths may have increased access to other sources of support, relying less on the specified protective factors. In our sample, Latinx and Hispanic youths were significantly more likely to live in households with lower caregiver education and income. Although socioeconomic status was controlled for in the analyses, residual effects may persist and partially explain observed differences.

Alternatively, limitations in data availability may have hindered fully capturing protective factors most relevant to non-Hispanic youths. It is possible that the ACEs that had the largest-magnitude associations with BMI, (ie, ACEs most responsive to self-coping or caregiver support) are less prevalent among non-Hispanic youths, leading to a smaller-magnitude association. However, our findings overall demonstrate that ethnicity influences associations of adversity and resiliency with weight, but further research is needed to better understand underlying mechanisms.

Altogether, these findings may have direct clinical implications. ACEs can be readily screened, and fostering protective factors may serve as a critical buffer against the effects of childhood adversity. For health care clinicians working with Latinx and Hispanic youths, integrating ACE assessments and protective factor evaluations into routine pediatric care may facilitate early identification of those at risk for obesity, enabling timely, targeted interventions. Interventions may be particularly beneficial in mitigating the effects of caregiver incarceration, which is more prevalent among Latinx and Hispanic youths than White non-Hispanic youths.^77^ Overall, bolstering self-coping skills and caregiver support may offer a promising pathway for resilience-based strategies aimed at reducing obesity risk and improving health outcomes among disproportionately impacted populations.

### Strengths and Limitations

This study had several strengths, including a large, sociodemographically diverse sample of youths not taking weight-impacting medications; in-person, objectively measured anthropometrics; self-reported protective factors; and a focus on Latinx and Hispanic youths. To our knowledge, this is the first study to simultaneously incorporate these features. Furthermore, the wide-ranging assessments of ACEs, with attention to both cumulative exposure and individual adversities, enabled a broad yet nuanced characterization of childhood adversity.

This study also has limitations. The study’s cross-sectional design and available variables restricted the ability to track BMI change or establish directionality of the association of ACEs with BMI. Potential bidirectional associations of individual ACEs with BMI cannot therefore be disentangled. Future ABCD data releases with increased longitudinal, in-person anthropometrics will allow examination of BMI trajectories and potential prediction of weight gain. Additionally, BMI is an imperfect marker for adiposity because it is limited in distinguishing fat distribution or lean mass, and may be less accurate in diverse populations.^78^ However, prior studies have demonstrated correlations between adiposity, lean mass, and BMI, and accordingly, it is often used as a screener.^79^ These analyses were constrained by data availability, limiting insight into metabolic risk beyond BMI.

ACE measurements were also restricted because questions did not capture all possible adversities. Moreover, wording, frequency of questioning, and respondent type (ie, caregiver vs youths) differed across questionnaires. Further research is needed to refine and standardize ACE assessment in clinical and research settings. While resilience is multifaceted, this study examined only 3 protective factors. Future work should incorporate longitudinal assessments of the association of ACEs with BMI and explore mechanistic pathways, such as systemic inflammation and stress-related psychopathology, which were beyond the scope of this study. Furthermore, while this research focused on Latinx and Hispanic youths, other groups are also disproportionately impacted by ACEs and obesity. Timely studies across diverse populations are needed to clarify these interactions and strengthen clinical and social interventions for broader populations.

## Conclusions

The pathways associating adversity with obesity are complex and multifactorial. The findings of this cross-sectional study suggest ACEs may be associated with increased BMI, and fostering resilience through protective factors may moderate some negative health outcomes linked to childhood adversity in Latinx and Hispanic youths. These findings highlight the critical role, particularly for health care clinicians, of incorporating early ACE screening to identify youths at risk for obesity while utilizing trauma-informed weight management strategies. It is important to consider how sociodemographic factors shape youths’ vulnerability to chronic stress and obesity and to promote resilience by bolstering self-coping skills and supportive adult relationships. These protective factors may buffer stress-induced weight gain and offer a promising path for personalized interventions. Advancing understanding of how adversity contributes to pediatric obesity can inform both medical and social interventions through targeted ACE assessments, promotion of protective factors, and stress-reduction strategies to lessen cortisol. Together, these efforts may help alter pediatric weight trajectories and improve health outcomes.

## Notes

Supplement 1. eAppendix. Literature Review and Search Parameters eTable 1. Literature Review eMethods. eTable 2. Number of Subjects Included at Each Criterion eTable 3. ACE Questions eTable 4. Protective Factor Questions eFigure 1. Weight Status Among Youth by Ethnicity eFigure 2. Distribution of Weight Status Across ACEs eResults. eTable 5. Impact of Protective Factors on ACE-BMI Relationship Stratified by Ethnicity eTable 6. Impact of Protective Factors on Individual ACE-BMI Relationship in Latinx/Hispanic Youth eTable 7. Impact of Protective Factors on Individual ACE-BMI Relationship in Non-Latinx/Hispanic Youth eReferences

Supplement 2. Data Sharing Statement
