# Supplementary material for: Adverse Experiences, Protective Factors, and Obesity in Latinx and Hispanic Youths
Source: JAMA Netw Open. 2025 Dec 4;8(12):e2547104. doi: 10.1001/jamanetworkopen.2025.47104 (PMC12679330; doi:10.1001/jamanetworkopen.2025.47104)
Supplement: Supplement 1. — eAppendix. Literature Review and Search Parameters eTable 1. Literature Review eMethods. eTable 2. Number of Subjects Included at Each Criterion eTable 3. ACE Questions eTable 4. Protective Factor Questions eFigure 1. Weight Status Among Youth by Ethnicity eFigure 2. Distribution of Weight Status Across ACEs eResults. eTable 5. Impact of Protective Factors on ACE-BMI Relationship Stratified by Ethnicity eTable 6. Impact of Protective Factors on Individual ACE-BMI Relationship in Latinx/Hispanic Youth eTable 7. Impact of Protective Factors on Individual ACE-BMI Relationship in Non-Latinx/Hispanic Youth eReferences [file jamanetwopen-e2547104-s001.pdf]

Supplemental Online Content

Goldman V, Esaian S, Rivas Fernández MÁ, et al. Adverse experiences, protective factors, and obesity in Latinx and Hispanic youths. *JAMA Netw Open*. 2025;8(12):e2547104. doi:10.1001/jamanetworkopen.2025.47104

eAppendix. Literature Review and Search Parameters

eTable 1. Literature Review

eMethods.

eTable 2. Number of Subjects Included at Each Criterion

eTable 3. ACE Questions

eTable 4. Protective Factor Questions

eFigure 1. Weight Status Among Youth by Ethnicity

eFigure 2. Distribution of Weight Status Across ACEs

eResults

eTable 5. Impact of Protective Factors on ACE-BMI Relationship Stratified by Ethnicity

eTable 6. Impact of Protective Factors on Individual ACE-BMI Relationship in Latinx/Hispanic Youth

eTable 7. Impact of Protective Factors on Individual ACE-BMI Relationship in Non-Latinx/Hispanic Youth

eReferences

This supplemental material has been provided by the authors to give readers additional information about their work.

© 2025 Goldman V et al. *JAMA Network Open*.

1. **eAppendix. Literature Review and Search Parameters**

1.1. **Literature Review**. PubMed search (9/11/25) on the topic of ACEs, childhood obesity and protective factors. This search yielded 749 studies published since 2000 (search terms listed below); a summary of the studies focused on ACEs, protective factors, and childhood obesity is provided in eTable1.

Search parameters:
("psychological trauma"[MeSH] OR ACE[tiab] OR ACES[tiab] OR "adverse childhood experiences"[MeSH] OR childhood-adversit[tiab] OR adversit*[tiab] OR childhoodtrauma*[tiab] OR adverse-childhood*[tiab] OR exposur*[tiab] OR childhood-experienc*[tiab] OR family-adversit*[tiab] OR trauma*[tiab] OR adverse-family-experienc*[tiab] OR adversefamily-event*[tiab] OR toxic*[tiab] OR toxic-stress*[tiab]) AND (obes*[tiab] OR overweight*[tiab] OR "obesity"[MeSH] OR "pediatric obesity"[MeSH Terms] OR childhood-obesit*[tiab] OR pediatric-obesit*[tiab] OR bmi[tiab] OR "body mass index"[MeSH] OR waist-circumferenc*[tiab] OR body-weight*[tiab] OR body-fat*[tiab] OR adiposit*[tiab] OR zscore*[tiab] OR "BMI percentil*"[tiab] OR overeat*[tiab] OR severe-obesit*[tiab]) AND ("child"[MeSH] OR "adolescent"[MeSH] OR "infant"[MeSH] OR "child, preschool"[MeSH] OR child*[tiab] OR adolescen*[tiab] OR teen*[tiab] OR infan*[tiab] OR baby[tiab] OR babies[tiab] OR kid[tiab] OR toddler*[tiab] OR preteen*[tiab] OR pediatric*[tiab] OR newborn*[tiab]) AND (protectiv*[tiab] OR buffer*[tiab] OR resilienc*[tiab] OR adaptiv*[tiab] OR coping[tiab] OR "coping strateg*"[tiab] OR "coping skill*"[tiab] OR supportive[tiab] OR "resilience, psychological"[MeSH] OR "adaptation, psychological"[MeSH] OR "coping"[MeSH])*

© 2025 Goldman V et al. *JAMA Network Open*.

eTable 1. Literature Review

Study	Dataset	N	Ages	% Latino/a/x/ Hispanic	Weight Measure	Weight Reporter	Protective Factors (PF)	PF reporter	Outcomes
**Foster, 2019[36]**	2016 National Survey of Children's Health (NSCH)[a]	24,405	10-17	Not reported[b]	Weight class groupings[c]: healthy weight [HW], overweight [OW], obese [OB])	Parent-reported	Family resilience, individual resilience	Parent	In higher-income groups only, ACEs linked to obesity; child resilience moderated this link, while family resilience did not.
**Lynch, 2018[10]**	2011-2012 NSCH	43,864	10-17	11.1%	Weight class groupings: underweight [UW]/HW, OW, OB	Parent-reported	Child resiliency, maternal mental health, parental engagement neighborhood safety	Parent	PF lowered OW/OB risk but did not significantly alter adversity effects.
**Bernard, 2019[37]**	Families with Child Protective Services (CPS) involvement	105	2-4	9%	Raw BMI	Measured	Attachment Style	Independent coder	Secure attachment was associated with lower BMI among children involved in CPS.
**Hall, 2021[38]**	Singular urban pediatric practice	443	12-18	21.1%	Weight class groupings: UW/HW/OW, OB	Measured	Child and youth resilience measure	Youth	ACE-BMI relationship was not moderated by protective factors. ACE-"poor health"[d] relationship was moderated by protective factors.

[a] NSCH database was used which over-sampled children with medical complexity and had higher responses among individuals who were from higher income brackets, outside metropolitan areas, college graduates, and non-Hispanic Caucasian.
[b] Not reported in overall sample; presented stratified by family-level income and weight status, with percentages only (no counts available).
[c] BMI percentiles were used for weight class groupings unless otherwise specified.
[d] Poor health" defined as having 1 or more: obesity, hypertension, depression.
[e] "Healthy weight behaviors": physical activity, sleep, screen time.
[f] BMI z-scores used for weight class groupings.

© 2025 Goldman V et al. *JAMA Network Open*.

Study	Dataset	N	Ages	% Latino/a/x/ Hispanic	Weight Measure	Weight Reporter	Protective Factors (PF)	PF reporter	Outcomes
**Hayes, 2021**[39]	NSCH	13,921	6-17	Not reported	Healthy Weight Behaviors[e]	Parent-reported	Family connection	Parent	Family connection moderated the role between healthy weight behaviors and adverse family experiences.
**Heerman, 2022**[18]	2016-2018 NSCH	30,023, 428	10-17	24.8%	Weight class groupings: UW/HW, OW, OB	Parent-reported	Family resilience	Parent	Family resilience moderated association between ACEs-BMI.
**Hall, 2023**[40]	Singular urban pediatric practice	414	6-11	Not reported	Weight class groupings: UW/HW/OW, OB	Measured	Child and Youth Resilience Measure	Parent	ACES and resilience did not predict obesity. Higher ACEs associated with behavioral symptoms, moderated by resilience.
**Covington, 2024**[41]	2017-2018 NSCH	24,100	10-17	25.7%	Weight class groupings: UW/HW, OW/OB	Parent-reported	Sleep duration	Parent	Increased sleep may moderate obesity risk, but only in adolescents without ACEs.
**Keane, 2024**[42]	2018-2020 NSCH	46,672	10-17	Not reported	Weight class groupings: UW/HW/OW, OB	Parent-reported	Parent and adult relationships, self-regulation	Parent	ACEs increased obesity risk; self-regulation, after-school activities and supportive neighborhood moderated link (except in those with ≥4 ACEs).

[a] NSCH database was used which over-sampled children with medical complexity and had higher responses among individuals who were from higher income brackets, outside metropolitan areas, college graduates, and non-Hispanic Caucasian.
[b] Not reported in overall sample; presented stratified by family-level income and weight status, with percentages only (no counts available).
[c] BMI percentiles were used for weight class groupings unless otherwise specified.
[d] Poor health" defined as having 1 or more: obesity, hypertension, depression.
[e] "Healthy weight behaviors": physical activity, sleep, screen time.
[f] BMI z-scores used for weight class groupings.

© 2025 Goldman V et al. *JAMA Network Open*.

Study	Dataset	N	Ages	% Latino/a/x/ Hispanic	Weight Measure	Weight Reporter	Protective Factors (PF)	PF reporter	Outcomes
**Mellar, 2025[43]**	Growing Up in New Zealand Study	4,895	8	Not reported	Weight class groupings[f]: UW/HW, OW/OB	Measured	Supportive relationships, positive environment, connections, socio-emotional competency.	Parent	Significant numbers of positive experiences may moderate ACE-obesity link.

[a] NSCH database was used which over-sampled children with medical complexity and had higher responses among individuals who were from higher income brackets, outside metropolitan areas, college graduates, and non-Hispanic Caucasian.
[b] Not reported in overall sample; presented stratified by family-level income and weight status, with percentages only (no counts available).
[c] BMI percentiles were used for weight class groupings unless otherwise specified.
[d] Poor health" defined as having 1 or more: obesity, hypertension, depression.
[e] "Healthy weight behaviors": physical activity, sleep, screen time.
[f] BMI z-scores used for weight class groupings.

© 2025 Goldman V et al. *JAMA Network Open*.

2. **eMethods**

2.1. **Study Design**. The ABCD® study started in 2016-2018 when the youth were 9-10-years-old. The data collection is anticipated to continue through 2025-2027 with participants reaching ages 19/20-years-old. Caregivers and youth provided written consent and assent in accordance with the centralized Institutional Review Board at the University of California San Diego. All 21 cites also received local IRB approval. Further information on ABCD® study design can be found at: www.ABCDStudy.org.

2.2. **Exclusion/Inclusion.** The ABCD enrollment exclusions included: history of major neurologic disorders, prematurity or specified birth complications, certain vision or hearing impairments, schizophrenia, severe autism, alcohol/substance use disorder, or intellectual disability. For this manuscript, we excluded youth 1) with invalid anthropometrics (e.g., weight changes exceeding expected normal variation [e.g., losing more than 50 pounds in one year], outliers identified through graphical visualization, remote visits due to Covid-19 pandemic); 2) taking medications impacting intake/weight (e.g., insulin, anti-hyperglycemic medications, stimulants, anti-psychotics, anti-depressants, weight loss medications); 3) with siblings (independence issues); or 4) missing key demographic, ACEs or protective factor data. See eTable 2 for breakdown of these exclusions.

2.3. **Demographics.** Race, as selected by the caregiver, had 19 options (Alaskan, American Indian, Asian Indian, Black/African American, Chinese, Filipino, Guamanian, Japanese, Korean, Native Hawaiian, Other Asian, Other Pacific Islander, Samoan, Vietnamese, White, other race, doesn't know, refuse to answer, and missing) and was classified into six categories: American Indian/Alaska Native/Native Hawaiian/Pacific Islander, Asian, Black, White, mixed, other. For the manuscript, we classified American Indian and Alaskan as "American Indian and Alaska Native (AIAN);" Asian Indian, Chinese, Filipino, Japanese, Korean, Vietnamese, other Asia as "Asian;" native Hawaiian, Guamanian, Samoan, other Pacific Islander as "Native Hawaiian and Other Pacific Islander (NHPI)." Mixed included endorsement of more than 1 race. "Other" was the endorsement by the caregiver. "Missing" was refuse to answer, doesn't know or missing. The authors acknowledge that these are simplifications of different types of ethnicities and races and do not capture the wide range of ethnic and racial identities but were used given the data availability in ABCD. Education was coded as highest obtained for caregivers across five categories (e.g., less than high school, high school diploma/GED, some college, bachelor's degree, post-graduate degree). Income was coded as "total combined family income" over 12 months and categorized as less than $49,999, $50,000-99,999, or greater than $100,000.

2.4. **Physical Data:**

2.4.1. **Puberty:** The Pubertal Developmental Scale (PDS)[1] is a sex-specific questionnaires completed at each visit. For boys, it asked about hair growth, voice change, and facial hair. For girls, it asked about body hair, breast development, and menarche. Possible responses included: not yet started (1 point), barely started (2 points), definitely started (3 points), seems complete (4 points) or missing. For boys, they are considered pre-puberty if the score is 3 points, early puberty 4-5 points, mid-puberty 6-8 points, late puberty 9-11 points, and post-puberty 12 points. For girls, no menarche is required for pre through mid-puberty, in addition to these point distributions: pre-puberty is 2 points, early puberty 3 points, mid-puberty greater than 3 points, late puberty less than or equal to 7 points with menarche, and post-puberty 8 points with menarche. To account for errors in scores by both adolescents (e.g., over or underestimated pubertal status based on relativity to peers) and adults (e.g., may be unaware of pubertal changes), averaged youth and caregiver scores were used to create a singular PDS score.[2,3]

2.4.2. **Anthropometrics:** Height and weight measurements were recorded by study personnel to the nearest 0.1 pound and 0.2 inch respectively. Assessments were repeated at least twice to enhance accuracy, and three times if there were any significant discrepancies. If two measurements were available, the average was taken. If three were available, the average was taken of the two closest to each other (i.e., presuming the third was the discrepant measurement). These measurements were utilized to calculate BMI. Raw, continuous BMI was used in the analyses for several reasons: 1) narrow age range (i.e., 11/12-years) allowing for minimal age-related differences; 2) BMI z-scores and percentiles were not developed from nationally representative populations (i.e., predominance non-Hispanic White youth, higher socioeconomic status) limiting accuracy for diverse populations;[4-7] and 3) future goal for longitudinal BMI research, for which BMI z-scores are not accurate (i.e., only accurate for cross-sectional analysis).[4] The Center for Disease Control and Prevention (CDC) sex-age-and-height specific BMI percentiles for weight classes and *z*-scores were used (eFigures 1).[5] Weight classes were used for clinical interpretation only, not main analyses. Youth with underweight

were included if their weight met other criteria (e.g., within 3 z-scores), since those in the lowest BMI percentiles (e.g., 1st, 2nd) reflect natural distribution of growth (i.e., in any sample, some youth will fall in the lowest percentiles), and do not necessarily indicate abnormality. This approach was taken to ensure completeness of the sample and comparability across the weight spectrum. Including youth with underweight also allowed us to examine whether ACEs were associated with deviations in weight in both directions, rather than only with elevated BMI. Outliers were excluded to minimize bias from data entry errors or underlying medical conditions.

2.5. **ACEs.** The total ACE score in this manuscript was generated from responses on 25 questions from eight questionnaires. This included ACEs that best represented the original ACEs from the CDC-Kaiser study[8] as well as prominent ACEs in the literature[9-18] including: bullying/ discrimination,[13,16] neighborhood violence,[10,11,17,19] and food/housing insecurity.[9-15] Neighborhood violence and socioeconomic hardship are particularly relevant for understanding the ACE-obesity relationship, given established links between neighborhood safety and obesity risk,[20] as well as between housing stability and ACEs.[21] The utilized questionnaires are described below. For questions asked across multiple years, some were initially phrased as "in your life" and later phrased as "within the past 12 months." To capture any incidence of ACEs, positive answers from any earlier time point were also used. For example, if the youth said "yes" to an ACE at baseline (asked about lifetime occurrence), but no at two-year follow up (asked about the past 12 months), it was coded as a "yes." Youth reported answers were prioritized when available. See eTable 3.

2.5.1. **Kiddie Schedule for Affective Disorders and Schizophrenia for School-Aged Children – Post-Traumatic Stress Disorder (KSADS PTSD)**. Semi-structured interview focused on symptoms and experiences contributing to psychiatric health challenges in youth.[22] Questions were drawn specifically from the PTSD portion. Administered at baseline and 2-year follow-up to caregivers only as binary (yes/no) questions.

2.5.2. **Life Events (PhenX).** Adverse Life Events Scale focuses on specific good and bad life events (e.g., injuries, new stepparent) and how much the event affected the youth.[23] Administered to youth and caregivers annually since 1-year follow-up, but only youth-answered binary (yes/no) questions were included.

2.5.3. **Demographics Survey**. Demographic information was collected from caregivers at baseline and follow-up years. This information was utilized for both general demographic information as well as ACEs regarding basic needs insecurity (e.g., unable to afford food, rent, utilities). Answers included "yes," "no," or refuse to answer (coded as missing data).

2.5.4. **Family Environment Scale (PhenX).** The Family Conflict subscale of the Family Environment Scale focuses on interpersonal relationships in families and openly expressed conflict.[24,25] Questions were asked annually, and dichotomous (true/false) youth responses were included.

2.5.5. **Discrimination Scale.** The Discrimination Scale[26] composed of questions from the 2006 Boston Youth Survey, with additional questions about discrimination frequency and experiences from the Measure of Perceived Discrimination. Only questions from the 2006 Boston Youth Survey about discrimination based on race, ethnicity, color, country of origin, or sexual identity were included. Questions were asked to youth during 1-year follow-up and 2-year follow-up and included answers of "yes," "no," "don't know," or refused to answer. Answers of "don't know" or refused to answer were coded as missing data.

2.5.6. **Cyberbullying.** Focuses on experiencing or perpetrating, frequency, and contributors to cyberbullying.[27] Youth-reported answers about experiencing cyberbullying were included. Answers were coded as "yes," "no," or refuse to answer (codded as missing data). Asked during 2-year follow-up to youth only.

2.5.7. **Neighborhood Safety/Crime Survey (PhenX).** This instrument is from the PhenX Neighborhood Safety Protocol that uses a 5-point Likert scale (e.g., 1= "strongly disagree," 5="strongly agree") to ask youth about the safety and crime in their neighborhood (i.e., 'area within about a 20-minute walk [or about a mile] from your home').[28] For the ACEs derived from this questionnaire, responses of "strongly agree" (=5), "agree" (=4), and "neutral" (=3) were coded as "yes" (i.e., not an ACE), while "strongly disagree" (=1) and "disagree" (=2) were coded as "no" (i.e., ACE). Questions were asked to youth at baseline, 1-year follow-up, and 2-year follow-up.

2.5.8. **Parental Monitoring.** This annually administered instrument assessed caregiver surveillance of children.[29-31] Youth-reported responses regarding their ability to contact a guardian when they are home alone were used. This instrument utilized a 5-point Likert scale (e.g., 1="never," 5="always or almost always"). For assessing ACE occurrence, responses of "always or almost always" (=5),

"often" (=4), and "sometimes" (=3) were coded as "yes" (i.e., not an ACE), while "never" (=1) and "almost never" (=2) were coded as "no" (i.e., ACE).

2.6. **Protective Factors.** The protective factor scores in this manuscript were derived from three questionnaires (e.g., peer health network, wills problem solving scale, and children's report of behavioral inventory). Each questionnaire represented one protective factor (see eTable 4).

2.6.1. **Wills Problem Solving Scale.** Focused on youth problem solving skills including gathering information, planning, exploration of alternatives, and overall thought process.[32] ABCD prioritized including questions that were associated with substance use behaviors in the literature. Self-control has been shown to contribute to resilience and buffer against certain stressors or risks.[32] Asked at 1-year follow up. Results comprised the first protective factor: self-coping skills.

2.6.2. **Children's Report of Parental Behavior Inventory (CRPBI).** An abbreviated version of the Acceptance Scale, a subscale of CRPBI, was administered to youth at baseline and 1-year follow-up.[33] The questions focus on youth perceptions of caregiver warmth, comfort, and openness.[34] Responses about primary caregiver were included in this manuscript. Answers were utilized for the second protective factor: caregiver support

2.6.3. **Peer Network Health:** Protective Scale. Collected youth reports on protective behaviors from three of their close friends. It has a particular focus on behaviors against substance use but asks varied questions regarding encouragement (e.g., 'encouraged you to get or stay involved with sports/exercise, school teams or clubs, volunteering, or religious activities').[35] It was asked at the 2-year follow-up. This comprised the third protective factor: friend support.

© 2025 Goldman V et al. *JAMA Network Open*.

eTable 2. Number of Subjects Included at Each Criterion

	n
Data available at the year 2 data frame	10973
Acceptable height range (within three z-scores)	10961
Valid height growth (e.g., did not shrink year to year)	10835
Acceptable weight range (within three z-scores)	10833
In-person height/weight measurement	9026
Not taking medication known to affect intake/weight	8044
Date available before Covid-19 pandemic	6627
Complete BMI data	6606
BMI within plausible range	6603
No siblings	5614
Complete demographic information (ethnicity, education)	5535
Complete ACE data	5435

Note. Data from year 2 was the focus for this manuscript, and as such the table reflects the number of youth with available data at this time point. Height and weight values were screened using *z*-scores from Centers for Disease Control and Prevention (CDC) growth charts.[5] Body mass index (BMI) was used as a continuous variable for analyses. ACE = adverse childhood experiences.

© 2025 Goldman V et al. *JAMA Network Open*.

eTable 3. ACE Questions

ACE	Questions	Years Asked	ABCD Assessment	Respondent
**Physical Abuse***	Shot, stabbed, or beaten brutally by a grown up in the home.	Baseline, Year2	KSADS PTSD	Caregiver
	Beaten to the point of having bruises by a grown up in the home.	Baseline, Year2	KSADS-PTSD	Caregiver
	Shot, stabbed, or beaten brutally by a non-family member.	Baseline, Year2	KSADS-PTSD	Caregiver
**Sexual Abuse***	A grown up in the home touched your child in their privates, had your child touch their privates, or did other sexual things to your child.	Baseline, Year2	KSADS-PTSD	Caregiver
	An adult outside your family touched your child in their privates, had your child touch their privates, or did other sexual things to your child.	Baseline, Year2	KSADS-PTSD	Caregiver
	A peer forced your child to do something sexually.	Baseline, Year2	KSADS-PTSD	Caregiver
**Emotional Abuse***	A family member threated to kill your child.	Baseline, Year2	KSADS-PTSD	Caregiver
**Neglect***	If you are at home when your parents or guardians are not, how often do you know how to get in touch with them?	Baseline, Year1, Year2	Parental Monitoring Survey [a]	Youth
**Family Member Substance Use***	Family member had drug and/or alcohol problem.	Year1, Year2	Adverse Life Events Scale	Youth
**Family Member Mental Health***	Family member had mental/emotional problem.	Year1, Year2	Adverse Life Events Scale	Youth
**Domestic Violence***	Witness the grownups in the home push, shove, or hit one another.	Baseline, Year2	KSADS-PTSD	Caregiver
	Family members sometimes hit each other.	Baseline, Year1, Year2	Family Environment Scale	Youth
**Divorce***	Parents separated or divorced?	Year1, Year2	Adverse Life Events Scale	Youth

Note. Questions were answered yes/no, unless marked otherwise. Refuse to answer and “don’t know” were coded as missing data. [a]Scale: 1=Never; 2=Almost Never; 3= Sometimes; 4= Often; 5= Always or Almost Always (reverse coded; answers 1 or 2 were scored as an ACE). *=Questions that aligned with original Kaiser-CDC Questionnaire.

© 2025 Goldman V et al. *JAMA Network Open*.

ACE	Questions	Years Asked	ABCD Assessment	Respondent
**Incarcerated Caregiver***	One of the parents/caregivers went to jail?	Year1, Year2	Adverse Life Events Scale	Youth
**Bullying**	In the past 12 months, have you felt discriminated against:<br>• Because of your race, ethnicity or color?<br>• Because you are (or your family is) from another country?<br>• Because someone thought you were gay, lesbian, or bisexual?	Year1, Year2	Discrimination Scale	Youth
	Have you been cyberbullied, where someone was trying on purpose to harm you or be mean to you online, in texts, or group texts, or on social media (like Instagram or Snapchat)?	Year2	Cyberbullying	Youth
**Basic Needs Insecurity**	In the past 12 months, has there been a time when you and your immediate family experienced any of the following:<br>• Needed food but couldn't afford to buy it or couldn't afford to go out to get it?<br>• Were without telephone service because you could not afford it?<br>• Didn't pay the full amount of the rent or mortgage because you could not afford it?<br>• Were evicted from your home for not paying the rent or mortgage?<br>• Had services turned off by the gas or electric company, or the oil company wouldn't deliver oil because payments were not made?	Baseline, Year1, Year2	Demographics	Caregiver
**Neighborhood Violence**	My neighborhood is safe from crime.	Baseline, Year1, Year2	Neighborhood Safety/Crime [b]	Youth
	Was a victim of crime/violence/assault?	Year1, Year2	Life Events	Youth

Note. Questions were answered yes/no, unless marked otherwise. Refuse to answer and "don't know" were coded as missing data. [b]Scale: 1=Strongly Disagree; 2=Disagree; 3= Neutral (neither agree nor disagree); 4= Agree; 5= Strongly Agree (answers 1 or 2 were scored as an ACE; reverse coded). *=Questions that aligned with original Kaiser-CDC Questionnaire.

© 2025 Goldman V et al. *JAMA Network Open*.

eTable 4. Protective Factors Questions

Protective Factors	Questions	ABCD Assessment
**Self-Coping Skills**	I get as much information as I can.	Wills Problem Solving Scale[a]
	I think hard about what steps to take.	
	I think about the choices before I do anything.	
	I think of different ways to take care of it.	
	I try different ways to solve the problem.	
	I do something to try to solve the problem.	
**Caregiver Support**	Makes me feel better after talking over my worries with him/her.	Children's Report of Parental Behavioral Inventory[b]
	Smiles at me often.	
	Is able to make me feel better when I am upset.	
	Believes in showing his/her love for me.	
	Is easy to talk to.	
**Friend Support**	1. During the last 6 months, have any of your close friends given you help with school, with money, with transportation, or help by talking through problems?<br>2. How much help did your close friends give you? Pick a number between 1 and 10.	Peer Network Health: Protective Scale[c]
	1. During the last 6 months, have any of your close friends encouraged you to get or stay involved with sports/exercise, school teams or clubs, volunteering, or religious activities?<br>2. How much help did your close friends encourage you? Pick a number between 1 and 10.	

Note. Protective Factors were gathered exclusively from youth reports. The Overall Protective Score was comprised as a mean of the three scores across each individual measure (e.g., self-coping skills, caregiver support, and friend support). [a]Scale: 1=Never; 2=A Little; 3= Sometimes; 4= Pretty Often; 5= Usually. [b]Scale: 1=Not like him/her; 2=Somewhat like him/her; 3=A lot like him/her. [c]Scale: Question 1: 0= No; 2 = Yes. If Yes, then Question 2: 1=1 – a little; 2=2; 3=3; 4=4; 5=5; 6=6; 7=7; 8=8; 9=9; 10=10 – lots.

© 2025 Goldman V et al. *JAMA Network Open*.

eFigure 1. Weight Status among Youth by Ethnicity

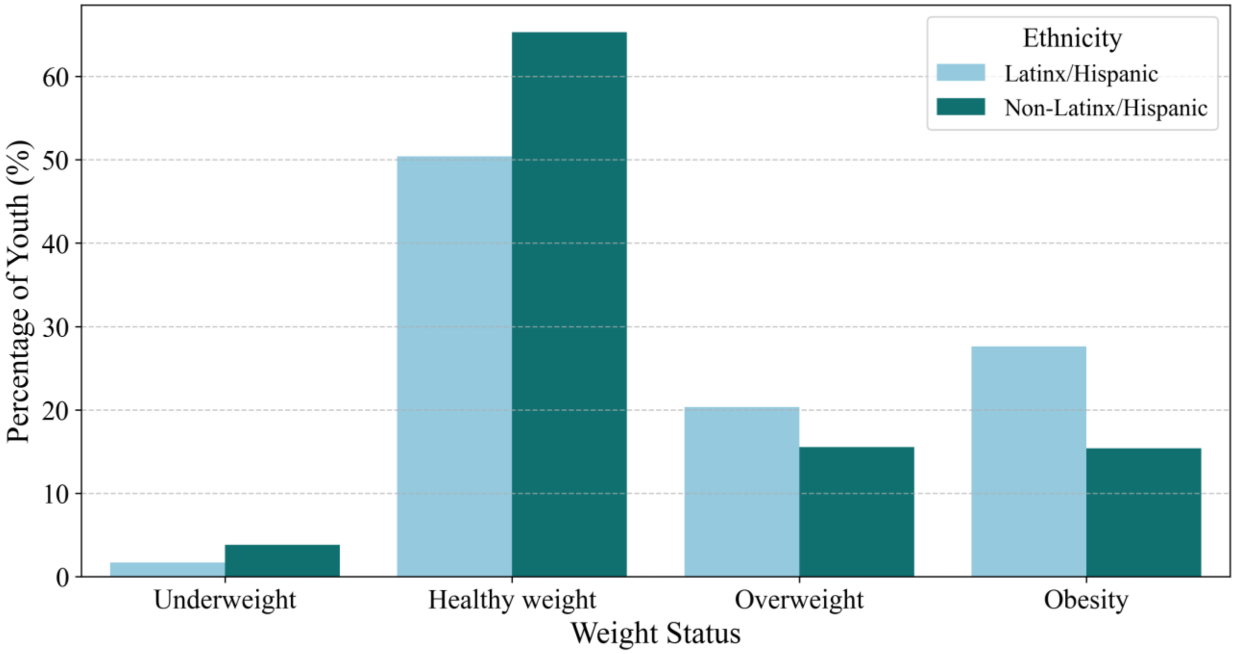

Note. Weight status categories, based on Centers for Disease Control and Prevention (CDC) body mass index (BMI) percentiles[5] (e.g., underweight < 5th %ile; healthy weight 5-84th %ile; overweight = 85th-94th %ile; obese >= 95th %ile), were applied only for clinical interpretation. BMI was used as a continuous variable for analyses. Ethnicity was reported by the caregiver as either "Latinx/Hispanic" (blue) or "non-Latinx/Hispanic" (green).

© 2025 Goldman V et al. *JAMA Network Open*.

eFigure 2. Distribution of Weight Status across ACEs

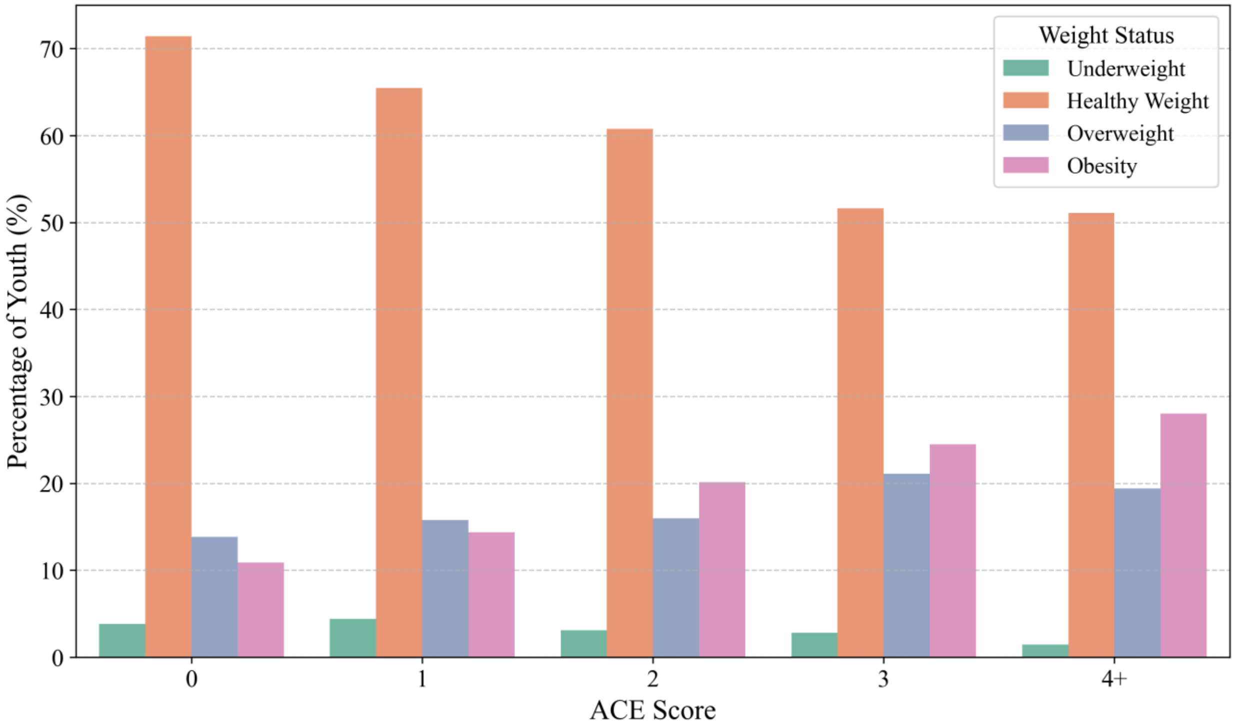

Note. Weight status categories, based on Centers for Disease Control and Prevention (CDC) growth charts using body mass index (BMI) percentiles[5] (e.g., underweight < 5th %ile [green]; healthy weight 5-84th %ile [orange]; overweight = 85th-94th %ile [blue]; obese >= 95th %ile [pink]), were applied only for clinical interpretation. BMI was used as a continuous variable for analyses. Total adverse childhood experience (ACE) score was collapsed into five categories for visualization purposes only. This classification was based on typical categorization in the literature: 0 ACEs, 1 ACE, 2 ACEs, 3 ACEs, and 4+ ACEs.

© 2025 Goldman V et al. *JAMA Network Open*.

3. eResults

3.1. **ACEs Prevalence.** In the sample, 62 youth reported physical abuse, 122 sexual abuse, 324 neglect, 41 emotional abuse, 1005 family member substance use, 861 family member mental health challenges, 2013 domestic violence, 1441 divorce, 983 neighborhood violence, 511 incarcerated parent, 1027 bullying, and 1272 basic needs insecurity. Weight status by ACEs score can be visualized in eFigure 2.

3.2. **ACE-Protective Factor-BMI Relationships.** Additional analyses were run testing pairwise combinations of protective factors: self-caregiver: (β=-0.762, p<0.001) and friend-self (β=-0.359, p=0.01) demonstrated significance, while friend-caregiver (β=-0.192, p=0.19) did not. This points towards protective associations being driven by self-coping skills. Two-way interactions between protective factors and total ACE score on BMI stratified by ethnicity as a sensitivity analysis are described in eTable 5. Two-way interactions between protective factors and individual ACEs on BMI in Latinx/Hispanic youth can be found in eTable 6 and in non-Latinx/Hispanic youth in eTable 7.

© 2025 Goldman V et al. *JAMA Network Open*.

eTable 5. Impact of Protective Factors on ACE - BMI Relationship Stratified by Ethnicity

Protective Factor	Latinx/Hispanic		Non-Latinx/ Hispanic	
	β	*p*	β	*p*
**Self-Coping Skills**	-0.648	**<0.001***	0.095	0.15
**Caregiver Support**	-0.341	**0.01***	0.033	0.59
**Friend Support**	-0.211	0.15	0.017	0.80
**Overall Score**	-0.500	**<0.001***	0.069	0.28

Note. In a sample stratified by ethnicity: Two-way interactions between total ACE Score and protective factors on BMI in Latinx/Hispanic youth and non-Latinx/Hispanic youth. Significant *p*-values (<0.05) are bolded; * indicates survived multiple comparison testing.

© 2025 Goldman V et al. *JAMA Network Open*.

eTable 6. Impact of Protective Factors on Individual ACE - BMI Relationship in Latinx/Hispanic Youth

	β	*p*		β	*p*
**Self – Coping Skills**			**Friend – Support**		
Physical Abuse	-0.207	0.92	Physical Abuse	-0.577	0.57
Sexual Abuse	-1.980	0.05	Sexual Abuse	0.450	0.64
Neglect	-0.078	0.89	Neglect	-0.040	0.94
Emotional Abuse	-1.101	0.69	Emotional Abuse	1.995	0.16
Family Substance Use	-0.855	**0.02**	Family Substance Use	-0.116	0.75
Family Mental Health	-1.061	**0.004**	Family Mental Health	0.135	0.73
Domestic Violence	-0.654	**0.03**	Domestic Violence	-0.395	0.18
Divorce	-0.739	**0.02**	Divorce	-0.263	0.38
Incarcerated Caregiver	-2.451	**<0.001***	Incarcerated Caregiver	-0.751	0.16
Bullying	-0.703	**0.04**	Bullying	-0.335	0.33
Basic Needs Insecurity	0.725	**0.02**	Basic Needs Insecurity	-0.243	0.41
Neighborhood Violence	0.192	0.56	Neighborhood Violence	-0.061	0.85
**Caregiver – Support**			**Overall Protect Score**		
Physical Abuse	-1.052	0.64	Physical Abuse	-0.711	0.60
Sexual Abuse	-2.677	0.05	Sexual Abuse	0.087	0.91
Neglect	0.204	0.62	Neglect	0.105	0.84
Emotional Abuse	-0.524	0.86	Emotional Abuse	1.268	0.23
Family Substance Use	-0.083	0.79	Family Substance Use	-0.320	0.38
Family Mental Health	-0.229	0.51	Family Mental Health	-0.323	0.38
Domestic Violence	-0.284	0.35	Domestic Violence	-0.088	0.76
Divorce	-0.691	**0.02**	Divorce	-0.312	0.27
Incarcerated Caregiver	-1.153	**0.01**	Incarcerated Caregiver	-1.496	**0.001***
Bullying	-0.650	0.05	Bullying	-0.432	0.18
Basic Needs Insecurity	-0.179	0.46	Basic Needs Insecurity	-0.086	0.76
Neighborhood Violence	-0.494	0.13	Neighborhood Violence	-0.309	0.33

Note. Two-way interactions between individual ACEs and protective factors on BMI in Latinx/Hispanic youth. Significant *p*-values (<0.05) are bolded; * indicates survived multiple comparison testing.

© 2025 Goldman V et al. *JAMA Network Open*.

eTable 7. Impact of Protective Factors on Individual ACE - BMI Relationship in non-Latinx/Hispanic Youth

	β	*p*		β	*p*
**Self – Coping Skills**			**Friend – Support**		
Physical Abuse	1.340	**0.007**	Physical Abuse	0.285	0.66
Sexual Abuse	1.118	**0.02**	Sexual Abuse	-0.075	0.87
Neglect	-0.766	**0.01**	Neglect	-0.144	0.70
Emotional Abuse	0.526	0.40	Emotional Abuse	0.258	0.77
Family Substance Use	-0.025	0.87	Family Substance Use	0.231	0.18
Family Mental Health	-0.134	0.49	Family Mental Health	0.064	0.73
Domestic Violence	0.226	0.11	Domestic Violence	-0.068	0.62
Divorce	0.036	0.82	Divorce	-0.136	0.38
Incarcerated Caregiver	0.548	**0.02**	Incarcerated Caregiver	-0.171	0.45
Bullying	0.213	0.24	Bullying	0.090	0.61
Basic Needs Insecurity	0.089	0.59	Basic Needs Insecurity	0.086	0.59
Neighborhood Violence	-0.104	0.56	Neighborhood Violence	0.187	0.28
**Caregiver – Support**			**Overall Protect Score**		
Physical Abuse	0.131	0.83	Physical Abuse	0.757	0.10
Sexual Abuse	0.191	0.67	Sexual Abuse	0.175	0.69
Neglect	-0.140	0.56	Neglect	-0.174	0.49
Emotional Abuse	0.919	0.15	Emotional Abuse	0.367	0.59
Family Substance Use	-0.185	0.25	Family Substance Use	0.077	0.66
Family Mental Health	-0.128	0.45	Family Mental Health	-0.047	0.80
Domestic Violence	-0.218	0.12	Domestic Violence	-0.030	0.83
Divorce	-0.003	0.98	Divorce	-0.043	0.78
Incarcerated Caregiver	0.308	0.14	Incarcerated Caregiver	0.175	0.42
Bullying	-0.018	0.91	Bullying	0.134	0.45
Basic Needs Insecurity	0.361	**0.02**	Basic Needs Insecurity	0.331	**0.03**
Neighborhood Violence	0.353	**0.03**	Neighborhood Violence	0.273	0.10

Note. Two-way interactions between individual ACEs and protective factors on BMI in non-Latinx/Hispanic youth. Significant *p*-values (<0.05) are bolded; None survived multiple comparison testing.

eREFERENCES:

1. Petersen AC, Crockett L, Richards M, Boxer A. A self-report measure of pubertal status: Reliability, validity, and initial norms. *J Youth Adolesc*. Apr 1988;17(2):117-33. doi:10.1007/bf01537962
2. Shirtcliff EA, Dahl RE, Pollak SD. Pubertal development: correspondence between hormonal and physical development. *Child Dev*. Mar-Apr 2009;80(2):327-37. doi:10.1111/j.1467-8624.2009.01263.x
3. Balzer BWR, Garden FL, Amatoury M, et al. Self-rated Tanner stage and subjective measures of puberty are associated with longitudinal gonadal hormone changes. *J Pediatr Endocrinol Metab*. Jun 26 2019;32(6):569-576. doi:10.1515/jpem-2019-0017
4. Adise S, Rhee KE, Laurent J, et al. Limitations of BMI z scores for assessing weight change: A clinical tool versus individual risk. *Obesity (Silver Spring)*. Mar 2024;32(3):445-449. doi:10.1002/oby.23957
5. Kuczmarski RJ, Ogden CL, Guo SS, et al. 2000 CDC Growth Charts for the United States: methods and development. *Vital Health Stat 11*. May 2002;(246):1-190.
6. Ryder JR, Kelly AS, Freedman DS. Metrics matter: Toward consensus reporting of BMI and weight-related outcomes in pediatric obesity clinical trials. *Obesity (Silver Spring, Md)*. 2022;30(3):571.
7. Jih J, Mukherjea A, Vittinghoff E, et al. Using appropriate body mass index cut points for overweight and obesity among Asian Americans. *Prev Med*. Aug 2014;65:1-6. doi:10.1016/j.ypmed.2014.04.010
8. Felitti VJ, Anda RF, Nordenberg D, et al. Relationship of childhood abuse and household dysfunction to many of the leading causes of death in adults. The Adverse Childhood Experiences (ACE) Study. *Am J Prev Med*. May 1998;14(4):245-58. doi:10.1016/s0749-3797(98)00017-8
9. Brieant A, Vannucci A, Nakua H, et al. Characterizing the dimensional structure of early-life adversity in the Adolescent Brain Cognitive Development (ABCD) Study. *Dev Cogn Neurosci*. Jun 2023;61:101256. doi:10.1016/j.dcn.2023.101256
10. Lynch BA, Finney Rutten LJ, Wilson PM, et al. The impact of positive contextual factors on the association between adverse family experiences and obesity in a National Survey of Children. *Prev Med*. Nov 2018;116:81-86. doi:10.1016/j.ypmed.2018.09.002
11. Heerman WJ, Krishnaswami S, Barkin SL, McPheeters M. Adverse family experiences during childhood and adolescent obesity. *Obesity (Silver Spring)*. Mar 2016;24(3):696-702. doi:10.1002/oby.21413
12. Isohookana R, Marttunen M, Hakko H, Riipinen P, Riala K. The impact of adverse childhood experiences on obesity and unhealthy weight control behaviors among adolescents. *Compr Psychiatry*. Nov 2016;71:17-24. doi:10.1016/j.comppsych.2016.08.002
13. Oh DL, Jerman P, Purewal Boparai SK, et al. Review of Tools for Measuring Exposure to Adversity in Children and Adolescents. *J Pediatr Health Care*. Nov-Dec 2018;32(6):564-583. doi:10.1016/j.pedhc.2018.04.021
14. Karcher NR, Niendam TA, Barch DM. Adverse childhood experiences and psychotic-like experiences are associated above and beyond shared correlates: Findings from the

© 2025 Goldman V et al. *JAMA Network Open*.

adolescent brain cognitive development study. *Schizophr Res*. Aug 2020;222:235-242. doi:10.1016/j.schres.2020.05.045
15. Smith KE, Pollak SD. Rethinking Concepts and Categories for Understanding the Neurodevelopmental Effects of Childhood Adversity. *Perspect Psychol Sci*. Jan 2021;16(1):67-93. doi:10.1177/1745691620920725
16. Stinson EA, Sullivan RM, Navarro GY, Wallace AL, Larson CL, Lisdahl KM. Childhood adversity is associated with reduced BOLD response in inhibitory control regions amongst preadolescents from the ABCD study. *Dev Cogn Neurosci*. Jun 2024;67:101378. doi:10.1016/j.dcn.2024.101378
17. Orendain N, Anderson A, Galván A, Bookheimer S, Chung PJ. A data-driven approach to categorizing early life adversity exposure in the ABCD Study. *BMC Med Res Methodol*. Jul 7 2023;23(1):164. doi:10.1186/s12874-023-01983-9
18. Heerman WJ, Samuels LR, González Peña T, et al. Family resilience and childhood obesity among children exposed to adverse childhood experiences in a national survey. *Obes Sci Pract*. Feb 2022;8(1):3-11. doi:10.1002/osp4.497
19. Warner TD, Leban L, Pester DA, Walker JT. Contextualizing Adverse Childhood Experiences: The Intersections of Individual and Community Adversity. *J Youth Adolesc*. Mar 2023;52(3):570-584. doi:10.1007/s10964-022-01713-2
20. Schroeder K, Schuler BR, Kobulsky JM, Sarwer DB. The association between adverse childhood experiences and childhood obesity: A systematic review. *Obes Rev*. Jul 2021;22(7):e13204. doi:10.1111/obr.13204
21. DeCandia CJ, Volk KT, Unick GJ. Evolving Our Understanding: Housing Instability as an ACE for Young Children. *Advers Resil Sci*. 2022;3(4):365-380. doi:10.1007/s42844-022-00080-y
22. Townsend L, Kobak K, Kearney C, et al. Development of Three Web-Based Computerized Versions of the Kiddie Schedule for Affective Disorders and Schizophrenia Child Psychiatric Diagnostic Interview: Preliminary Validity Data. *J Am Acad Child Adolesc Psychiatry*. Feb 2020;59(2):309-325. doi:10.1016/j.jaac.2019.05.009
23. Tiet QQ, Bird HR, Hoven CW, et al. Relationship between specific adverse life events and psychiatric disorders. *J Abnorm Child Psychol*. Apr 2001;29(2):153-64. doi:10.1023/a:1005288130494
24. Moos RH, Moos, B.S. . Family Environment Scale Manual. *Consulting Psychologists Press, Palo Alto, CA*. 1994;
25. Boyd CP, Gullone E, Needleman GL, Burt T. The Family Environment Scale: reliability and normative data for an adolescent sample. *Fam Process*. Dec 1997;36(4):369-73. doi:10.1111/j.1545-5300.1997.00369.x
26. Borges G, Azrael D, Almeida J, et al. Immigration, suicidal ideation and deliberate self-injury in the Boston youth survey 2006. *Suicide Life Threat Behav*. Apr 2011;41(2):193-202. doi:10.1111/j.1943-278X.2010.00016.x
27. Stewart RW, Drescher CF, Maack DJ, Ebesutani C, Young J. The Development and Psychometric Investigation of the Cyberbullying Scale. *J Interpers Violence*. Aug 2014;29(12):2218-2238. doi:10.1177/0886260513517552

© 2025 Goldman V et al. *JAMA Network Open*.

28. Mujahid MS, Diez Roux AV, Morenoff JD, Raghunathan T. Assessing the measurement properties of neighborhood scales: from psychometrics to ecometrics. *Am J Epidemiol*. Apr 15 2007;165(8):858-67. doi:10.1093/aje/kwm040
29. Stattin H, Kerr M. Parental monitoring: a reinterpretation. *Child Dev*. Jul-Aug 2000;71(4):1072-85. doi:10.1111/1467-8624.00210
30. Chilcoat HD, Anthony JC. Impact of parent monitoring on initiation of drug use through late childhood. *J Am Acad Child Adolesc Psychiatry*. Jan 1996;35(1):91-100. doi:10.1097/00004583-199601000-00017
31. Karoly HC, Callahan T, Schmiege SJ, Ewing SW. Evaluating the Hispanic Paradox in the Context of Adolescent Risky Sexual Behavior: The Role of Parent Monitoring. *J Pediatr Psychol*. May 2016;41(4):429-40. doi:10.1093/jpepsy/jsv039
32. Wills TA, Ainette MG, Stoolmiller M, Gibbons FX, Shinar O. Good self-control as a buffering agent for adolescent substance use: an investigation in early adolescence with time-varying covariates. *Psychol Addict Behav*. Dec 2008;22(4):459-71. doi:10.1037/a0012965
33. Schaefer ES. A configurational analysis of children's reports of parent behavior. *J Consult Psychol*. Dec 1965;29(6):552-7. doi:10.1037/h0022702
34. Barber BK, Olsen JE, Shagle SC. Associations between parental psychological and behavioral control and youth internalized and externalized behaviors. *Child Dev*. Aug 1994;65(4):1120-36.
35. Mason M, Light J, Campbell L, et al. Peer Network Counseling with Urban Adolescents: A Randomized Controlled Trial with Moderate Substance Users. *J Subst Abuse Treat*. Nov 2015;58:16-24. doi:10.1016/j.jsat.2015.06.013
36. Foster BA, Weinstein K. Moderating Effects of Components of Resilience on Obesity Across Income Strata in the National Survey of Children's Health. *Acad Pediatr*. Jan-Feb 2019;19(1):58-66. doi:10.1016/j.acap.2018.08.012
37. Bernard K, Frost A, Jelinek C, Dozier M. Secure attachment predicts lower body mass index in young children with histories of child protective services involvement. *Pediatr Obes*. Jul 2019;14(7):e12510. doi:10.1111/ijpo.12510
38. Hall A, Perez A, West X, et al. The Association of Adverse Childhood Experiences and Resilience With Health Outcomes in Adolescents: An Observational Study. *Glob Pediatr Health*. 2021;8:2333794x20982433. doi:10.1177/2333794x20982433
39. Hayes K, Davis A, Zlomke K, Fruh S. Importance of Family Connection for Healthy Weight Behaviors Amid Adverse Family Experiences and Behavioral Difficulties. *J Dev Behav Pediatr*. Dec 1 2021;42(9):e1-e7. doi:10.1097/dbp.0000000000000954
40. Hall A, West X, Brown M, et al. Association of Adverse Childhood Experiences and Resilience With Obesity, High Blood Pressure, and Parental Report of Behavioral Health Symptoms in Children: A Cross Sectional Study. *Glob Pediatr Health*. 2023;10:2333794x231159518. doi:10.1177/2333794x231159518
41. Covington LB, Ji X, Brownlow JA, Ji M, Patterson F. Adverse Childhood Experiences May Dampen the Protective Role of Sleep Duration on Adolescent Obesity Risk. *J Community Health*. Oct 2024;49(5):809-819. doi:10.1007/s10900-024-01344-7
42. Keane K, Evans RR, Wilkinson LL, King DM, Leban L, Macrina D. Adverse Childhood Experiences, Protective Factors, and Childhood Obesity: Comparing the Effectiveness of

© 2025 Goldman V et al. *JAMA Network Open*.

Three Resilience Frameworks. *Adversity and Resilience Science*. 2024/09/18 2024;doi:10.1007/s42844-024-00151-2
43. Mellar BM, Ghasemi M, Gulliver P, et al. Identification of positive childhood experiences with the potential to mitigate childhood unhealthy weight status in children within the context of adverse childhood experiences: a prospective cohort study. *BMC Public Health*. Jan 13 2025;25(1):8. doi:10.1186/s12889-024-20727-y

© 2025 Goldman V et al. *JAMA Network Open*.
