# Supplementary material for: Adverse Experiences, Protective Factors, and Obesity in Latinx and Hispanic Youths
Source: JAMA Netw Open. 2025 Dec 4;8(12):e2547104. doi: 10.1001/jamanetworkopen.2025.47104 (PMC12679330; doi:10.1001/jamanetworkopen.2025.47104)
Supplement: Supplement 2. — Data Sharing Statement [file jamanetwopen-e2547104-s002.pdf]

Data Sharing Statement

Goldman. Adverse Experiences, Protective Factors, and Obesity in Latinx and Hispanic Youths. *JAMA Netw Open*. Published December 04, 2025.
doi:10.1001/jamanetworkopen.2025.47104

Data
Data available: No

Additional Information
Explanation for why data not available: The data used in the preparation of this article were obtained from ABCD® (https://abcdstudy.org/), 5.0 release (DOI: 10.15154/z563-zd24), which is held in the NIMH Data Archive (NDA). A data use agreement is needed to utilize this public data. Analytical code available at: github.com/Adise-lab/ABCD_ACES_PF_ BMI.
